# Cross-Dataset Evaluation of an Automated Video-Based Model for Detecting Tardive Dyskinesia Using the Clinician’s Tardive Inventory: Validation Study

**DOI:** 10.2196/92197

**Published:** 2026-05-14

**Authors:** Richard Trosch, Anthony Sterns, Bradley Grimm, Loren Larsen, Bretton Talbot, Fred Ma, Rakesh Ranjan, Carlene MacMillan, Joel Hughes, Joseph Friedman, Owen Muir

**Affiliations:** 1 William Beaumont School of Medicine Oakland University Farmington, MI United States; 2 Scripps Gerontology Center Miami University Oxford, OH United States; 3 Videra Health Orem, UT United States; 4 iRxReminder Cleveland, OH United States; 5 Charak Health and Wellness Center Cleveland, OH United States; 6 Radial Health Brooklyn, NY United States; 7 Department of Psychological Sciences Kent State University Kent, OH United States; 8 Butler Hospital Providence, RI United States

**Keywords:** mental health, artificial intelligence, AI, medication adherence, tardive dyskinesia, remote patient monitoring, movement disorders, Clinician’s Tardive Inventory, CTI, Abnormal Involuntary Movement Scale, AIMS

## Abstract

**Background:**

Tardive dyskinesia (TD) is a common, often underrecognized movement disorder resulting from long-term antipsychotic use, yet its detection in routine mental health care remains inconsistent despite the availability of structured rating scales.

**Objective:**

This study evaluated the performance of an artificial intelligence–powered, video-based model for detecting abnormal movements associated with TD using the Clinician’s Tardive Inventory (CTI) dataset. We compare automated assessments of videos from the CTI dataset with previously completed clinician-rated Abnormal Involuntary Movement Scale (AIMS) and CTI scores for the dataset’s videos to determine the model’s reliability and the accuracy of its assessment conclusions relative to expert raters.

**Methods:**

In total, 69 videos with corresponding AIMS and CTI ratings were analyzed using the visual transformer algorithm model called TDtect reported previously. The dataset included single-video assessments per participant, with varied instructions and movement types. The relationship between automated predictions and clinician ratings was assessed using Pearson correlation, and predictive accuracy was evaluated using area under the curve (AUC) metrics.

**Results:**

The model showed a strong correlation with AIMS total scores (*r*=0.717) and high diagnostic accuracy (AUC 0.854), which improved further at an optimized threshold (AUC 0.900). Performance differed across anatomical regions, with the tongue, lips, and jaw displaying the highest predictive reliability. Functional CTI components had weaker correlations (*r*=0.27-0.63), as expected due to the subjective nature of these measures.

**Conclusions:**

These findings provide preliminary evidence that an artificial intelligence–driven TD detection model can generalize across video protocols, suggesting potential for broader clinical applicability, although further validation is needed. Future refinements and fine-tuning are expected to enhance accuracy, particularly in predicting functional impact.

## Introduction

Tardive dyskinesia (TD) is a persistent neurological disorder caused by exposure to dopamine receptor–blocking agents, including antipsychotics and antiemetics [1]. It is characterized by symptoms including stereotypy, akathisia, dystonia, chorea, myoclonus, vocalizations, and sensory issues [2]. The prevalence of TD among those exposed is estimated to range from 15% to 40% in the United States, with risk factors such as age, drug class, treatment duration, and the presence of movement-related side effects during early treatment stages [3-6]. In addition to discomfort, TD is linked to increased morbidity, stigmatization, and reduced quality of life [7].

Despite available treatments, TD remains underdiagnosed [8]. Early detection of TD facilitates prompt treatment with effective interventions to mitigate morbidity. The gap in the use of the 2 available Food and Drug Administration–designated breakthrough treatments, compared to the predicted prevalence of TD in the population, highlights the need for improved screening methods.

Traditional TD screening and assessment rely on clinician-administered tools such as the Abnormal Involuntary Movement Scale (AIMS). However, the AIMS is a rating scale designed to quantify the severity of abnormal movements across 7 anatomical regions, and its use to screen for TD may be inappropriate according to expert consensus [9]. Furthermore, the AIMS has notable limitations [10,11]. It lacks specific movement descriptors, does not comprehensively address all TD movements, does not document the number of abnormal movements present, and does not provide independent measures of frequency and amplitude. As our understanding of TD phenomenology has expanded, there is a need for updated and objective diagnostic tools [12]. The Clinician’s Tardive Inventory (CTI) is a validated tool developed to address these limitations, integrating both structured anatomical movement assessment and evaluation of functional impact into a single, repeatable inventory.

Sterns et al [13] described the TDtect visual transformer algorithm, a novel deep learning model designed to enhance the accuracy of TD detection using automated video analysis. Although traditional convolutional neural networks [13,14] excel at extracting local spatial features, TDtect leverages a transformer architecture to process video frames by dividing them into patches and using self-attention mechanisms to capture long-range temporal dependencies across movements. This approach enables more comprehensive feature extraction across the temporal dimension.

The initial studies on TDtect demonstrated improved sensitivity and specificity in identifying TD-related motor disturbances compared to clinician-rated AIMS measures. By leveraging machine learning, TDtect may allow reduced dependence on prior clinical experience in routine TD screening. TDtect is designed to be deployed through a smartphone app, at a scale commensurate with the millions of individuals likely affected.

The initial studies evaluating the TDtect algorithm demonstrated that visual transformer algorithms could be used to analyze facial, oral, and body movements in video recordings of AIMS assessments. However, the initial investigations only evaluated this algorithm on videos in which participants performed the steps of the AIMS assessment. This study evaluates the model’s performance against a different dataset. Here, we evaluate a video dataset collected to evaluate the clinician-rated CTI. This collection of 69 patient videos, with corresponding expert-completed ratings on both CTI and AIMS, was used as an additional independent dataset to evaluate the performance of the TDtect visual transformer model.

## Methods

### Dataset and Participants

The CTI dataset initially contained 73 video recordings of people, many of whom are living with TD. Of these, 70 had corresponding AIMS and CTI ratings. After matching videos with their appropriate ratings, we obtained 69 complete samples (video recordings with matched AIMS and CTI ratings) for our final analysis. Two movement disorders specialists independently confirmed the diagnosis of TD [12]. Unlike structured National Institute of Health datasets, which use multiple reviewers and standardized movement instructions, this dataset included single-video assessments with adaptive participant instructions. The videos ranged from 1 to 6 minutes in length and included individuals sitting, standing, and performing targeted movements as shown in Table 1.

**Table 1:** Description of the video data components for each study element.

Study element	Participants, n (%)^a^	Videos, n	Clip duration (seconds), mean (SD)	Session duration (minutes), mean (SD)	Assessment
Study 1^b^	46 (100)	18	34 (19)	11.40 (2.98)	AIMS^c,d^
Study 2^b^	136 (100)	22	47 (31)	13.50 (3.77)	AIMS^d^
Study 3^b^	174 (100)	4	71 (50)	3.43 (2.11)	AIMS^e^
CTI^f^ dataset	69 (100)	1	170 (71)	2.83 (1.19)	AIMS+CTI^g^

^a^Participant n values reflect independent study populations enrolled under separate protocols; aggregate percentages across studies are not reported as they would be misleading.

^b^Studies 1 to 3 from the iRx-Videra National Institute of Health protocol (Sterns et al [13]).

^c^AIMS: Abnormal Involuntary Movement Scale.

^d^Assessment was conducted using recorded videos in a controlled environment.

^e^Assessment was performed by a clinician in a face-to-face clinical setting.

^f^CTI: Clinician’s Tardive Inventory.

^g^Assessments were performed by a clinician in a face-to-face clinical setting while being recorded.

### Video Processing and Analysis

Video recordings from the CTI dataset were processed using a standardized pipeline. First, we used facial detection algorithms to isolate the face, which was analyzed separately from the body before combining the features later in our pipeline. Then, the model examined movement patterns at the per-second level using temporal feature extraction to identify potential dyskinetic movements. For each video, multiple regions of interest (facial and full body) were processed independently to ensure comprehensive movement detection. The final severity scores were derived from aggregated regional assessments, with automated quality control algorithms excluding segments with poor video quality or tracking failures. Regional movement frequencies and amplitudes were quantified using statistical features that capture temporal patterns characteristic of TD movements. This approach enabled assessment of both overall TD severity and anatomical distribution without requiring manual preprocessing or standardized instructions.

### The CTI Tool

The CTI was developed as a structured assessment tool to document anatomical movement abnormalities and functional impairments associated with TD. The scale evaluates six core movement categories:

1. Eye, eyelid, and facial movements (eg, excessive blinking, forceful eyelid closure, and facial grimacing)
2. Tongue and mouth movements (eg, tongue darting, lip smacking, and intraoral movements)
3. Jaw movements (eg, chewing, grinding, and jaw displacement)
4. Limb and trunk movements (eg, repetitive foot tapping, truncal rocking, and postural adjustments)
5. Complex movements (eg, hand-wringing, self-caressing, and fidgety leg movements)
6. Abnormal vocalizations (eg, irregular respiration, grunting, and moaning)

Each movement category is rated on a 0 to 3 scale based on frequency, with scores ranging from absent (0) to constant (3). Functional impairments, including activities of daily living, social distress, and physical harm, are rated separately from 0 (unaware or unaffected) to 3 (severe impact) [12].

### TDtect Algorithm

The development of the TDtect algorithm has been described previously by Sterns et al [13]. The current TDtect model incorporates refinements since the original publication, including enhanced capability to process unstructured video assessments of variable duration through temporal averaging of frame-level predictions. These improvements enable the model to maintain diagnostic accuracy across diverse clinical video protocols without requiring standardized assessment instructions. In brief, the TDtect training dataset consisted of 3979 video responses collected across 3 independent studies involving 356 participants. Study 1 (46/356, 13%) and study 2 (136/356, 38%) were video AIMS assessments conducted in a clinical setting and evaluated by 3 trained raters. Study 3 (174/356, 49%) consisted of patients with a traditional AIMS assessment evaluated by a single trained rater. The TDtect video assessment session has four segments: (1) the patient sitting still, (2) the patient with an open mouth and tongue inside, (3) the patients with an open mouth and the tongue extended, and (4) answering an open-ended question about daily activities. The assessments had an average duration of 2 minutes and 44 seconds.

The TDtect algorithm architecture model uses a vision transformer framework, which enables precise tracking of subtle, involuntary movements. TDtect’s performance was previously benchmarked against clinician-rated AIMS with an area under the curve (AUC) of 0.89 in validation testing [13].

The TDtect model was not trained on any of the CTI dataset used to validate the CTI [12]. However, given the shared clinical population of individuals with TD in the CTI dataset, it was deemed a valid use of the TDtect model. The TDtect model has continued to be refined since the algorithm was reported [13]. This study used the most current iteration of the algorithm, trained through December 2024, which incorporates more participants and enhanced labeling methodologies. These enhancements improved the AUC from 0.89 to 0.906 [13]. Performance metrics have improved on validation datasets compared to earlier versions. A key enhancement is its expanded capability for analyzing unstructured assessment sessions, allowing the model to evaluate TD movements regardless of specific instruction protocols. The model architecture has also been optimized to better handle the variability in real-world clinical assessments while maintaining high diagnostic accuracy. Detailed technical specifications and comparative performance metrics of this refined algorithm will be addressed in a separate technical publication.

### Statistical Analysis

For this cross-dataset evaluation, we analyzed 69 paired samples of video recordings and corresponding clinical ratings from the CTI dataset. The statistical analysis plan designated two primary outcomes: (1) Pearson correlation coefficient between automated TDtect scores and clinician-rated AIMS total scores and (2) area under curve (AUC) or more formally area under the receiver operating characteristic curve, for TD diagnosis using the Schooler-Kane criteria [15]. Correlation between TDtect regional subscores and CTI frequency ratings was examined as an exploratory secondary analysis to assess anatomical specificity.

For the AUC analysis, we used 2 complementary approaches. First, we measured AUC using the Schooler-Kane methodology for TD diagnosis [15] as the reference standard. However, due to the high prevalence of positive cases in our population, which created a significant class imbalance, we also evaluated AUC using a more stringent threshold to better differentiate between moderate and severe cases. This second approach allowed us to assess the model’s ability to identify pronounced TD cases, which may be more clinically relevant. Sensitivity and specificity were calculated for both ground-truth labeling schemes, with the optimal model output cut point for each determined using Youden J statistic [16]. As the Youden-optimal cut point was identified on the same dataset used for evaluation, reported sensitivity and specificity values should be considered exploratory; AUC, which integrates performance across all thresholds, is the primary performance metric. This comprehensive approach enables assessment of both continuous score agreement and categorical diagnostic performance across different severity levels.

### Ethical Considerations

The cited study by Sterns et al [13], on which the algorithm is based, was approved and overseen by the Creative Action LLC Institutional Review Board, a National Institutes of Health–registered institutional review board (FWA #10005). All patients provided informed consent, which was documented and reviewed. Participants’ video data were only available to the expert reviewers. The participants received compensation in the form of a gift card.

## Results

### Overall Model Performance

The TDtect model, without any retraining or fine-tuning for assessment of the CTI dataset, demonstrated a strong correlation with clinician-rated AIMS total scores (*r*=0.717; 69/69, 100%). This correlation strength is particularly notable given the nonstandardized nature of the video protocol used in the CTI dataset compared to the structured protocol used in the model’s original training.

**Figure 1:**
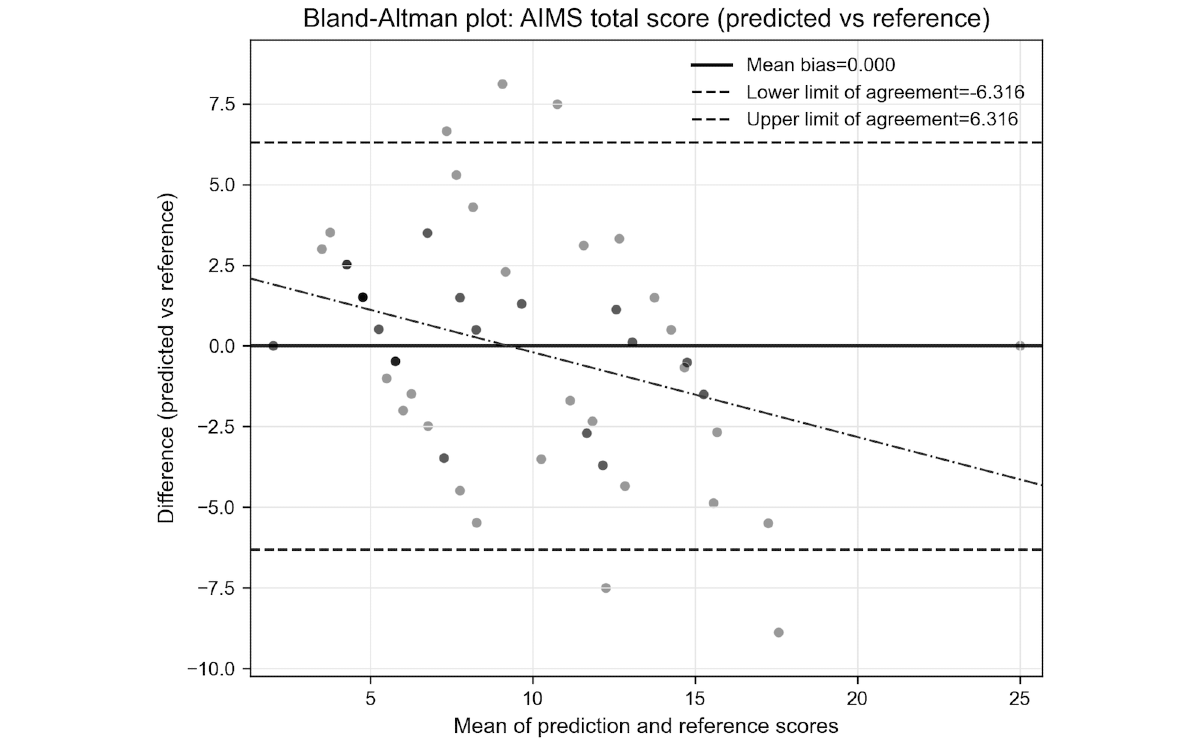
Bland-Altman graph. AIMS: Abnormal Involuntary Movement Scale.

Bland-Altman analysis was performed using isotonic regression calibration to assess score-level agreement, as the unstructured assessment version produces uncalibrated outputs that require alignment with clinician rating scales (Figure 1) [17]. The analysis demonstrated negligible bias (mean difference approximately 0), with limits of agreement ranging from –6.32 to +6.32. A small but statistically significant proportional bias remained (slope=–0.26; *P=*.002), suggesting slight underestimation at higher severity levels.

**Figure 2:**
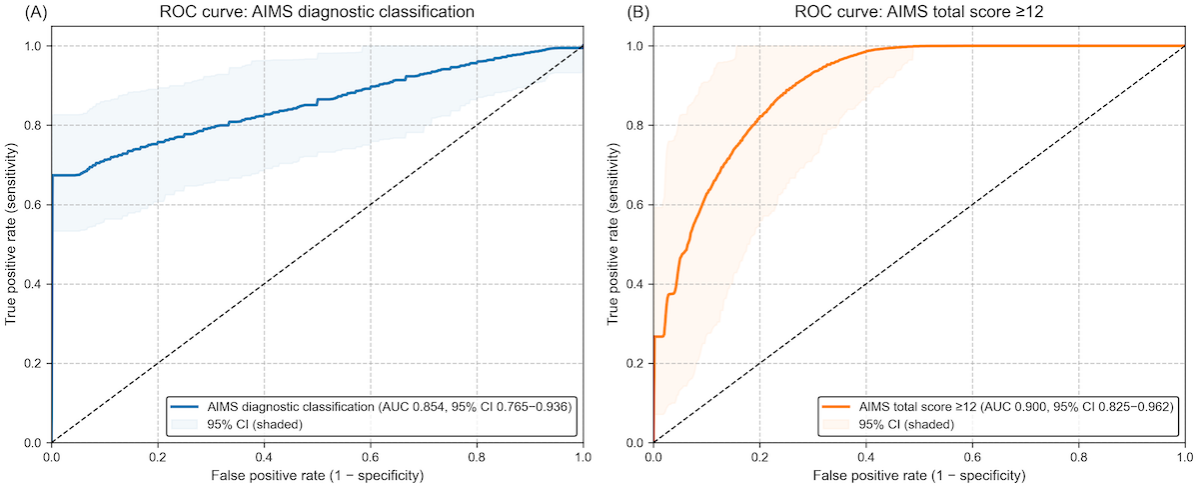
Receiver operating characteristic (ROC) curves for model performance, showing (A) discrimination for the Abnormal Involuntary Movement Scale (AIMS) diagnostic classification and (B) discrimination for AIMS total scores ≥12.

Diagnostic classification performance was evaluated using receiver operating characteristic curve analysis. Using the Schooler-Kane diagnostic criteria for TD identification, the model achieved an AUC of 0.854 in a dataset in which 80% of samples showed positive findings according to clinician rating [15]. This performance is comparable to the AUC of 0.89 reported in our previous validation studies using standardized assessment protocols (Figure 2) [13]. Table 2 summarizes the key performance metrics of the model, including correlation with AIMS, diagnostic accuracy measures, and characteristics of the dataset used for validation. Calibration analysis yielded a Brier score of 0.137 (AIMS ≥12), and the calibration curve (Multimedia Appendix 1) showed reasonable calibration across the mid- to high-probability range.

**Table 2:** Model performance metrics for predicting Abnormal Involuntary Movement Scale (AIMS) scores (N=69).

Metric		Values
Pearson correlation with AIMS (95% CI)		0.717 (0.577-0.824)
Diagnostic AUC (95% CI)^a,b^		0.854 (0.754-0.934)
AUC (95% CI) (threshold ≥12)		0.900 (0.822-0.958)
Sensitivity (95% CI)		0.727 (0.566-0.875)
Specificity (95% CI)		0.929 (0.818-1.000)
Brier score (Schooler-Kane criteria)^c^		0.128
Brier score (AIMS ≥12)^c^		0.137
Positive cases (%)		80%
**Video duration (seconds)**		
	Range	34-388
	Mean (SD)	170 (71)

^a^AUC: area under the curve.

^b^Diagnostic classification was based on the Schooler-Kane criteria.

^c^For reference, a naive classifier predicting the base rate for every sample would give a Brier score of approximately 0.16 (Schooler-Kane criteria) and approximately 0.23 (AIMS ≥12); therefore, the model outperformed that baseline.

**Figure 3:**
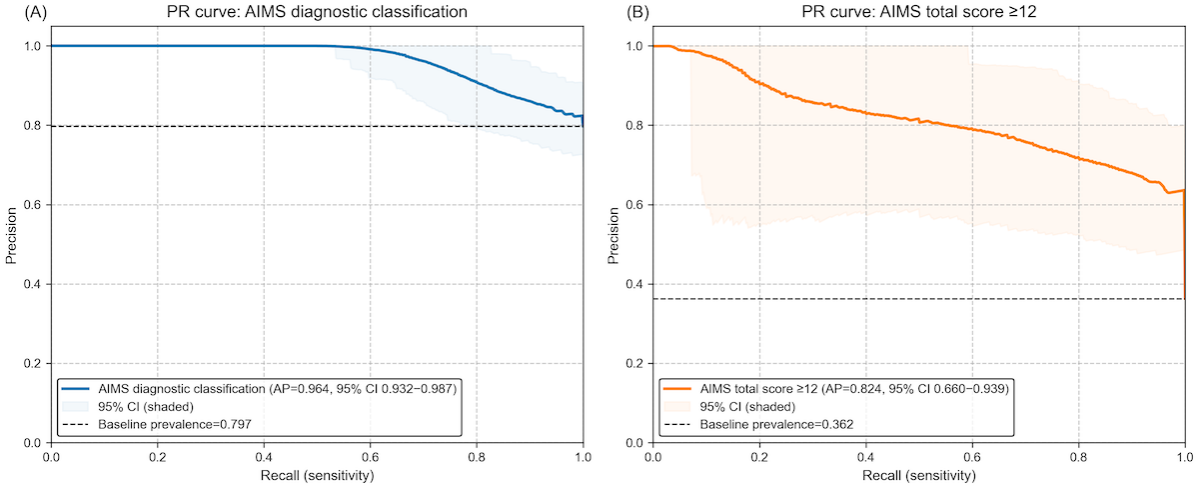
Precision-recall (PR) analysis for TDtect model performance, showing (A) discrimination for Abnormal Involuntary Movement Scale (AIMS) diagnostic classification and (B) discrimination for AIMS total scores ≥12. AP: average precision.

Using the Schooler-Kane criteria, TDtect achieved average precision scores of 0.964 (Figure 3A) and 0.822 for AIMS ≥12 (Figure 3B). The precision-recall curves show model performance across all decision thresholds, with precision representing positive predictive value and recall representing sensitivity. The baseline represents the proportion of positive cases in each classification scheme (79.7%, 55/69 and 36.2%, 25/69, respectively). These results complement the receiver operating characteristic analysis and provide additional insight into model performance for clinical screening applications where positive predictive value is particularly important.

When analyzing optimal threshold selection, we found that classification performance could be further improved to an AUC of 0.900 when using a more stringent threshold (AIMS total score≥12), which identified 36% of the sample as positive cases. This finding suggests that the model demonstrates particularly high accuracy in identifying more pronounced cases of TD, which has important clinical implications for identifying patients most in need of intervention [18].

### Anatomical Region-Specific Performance

The model’s detection capabilities varied across different anatomical regions, providing insights into its strengths and potential limitations in clinical application. Figure 2 summarizes the AUC values across different body regions and severity thresholds.

The orofacial region, particularly tongue movements, demonstrated the highest detection reliability (AUC range 0.87-0.95 across severity thresholds). This aligns with clinical expectations, as abnormal tongue movements represent one of the most visually distinctive and prevalent manifestations of TD. Lip and jaw movements also showed good detection performance (AUC 0.76-0.92), consistent with the orofacial predominance typically seen in TD.

Upper extremity movements were detected with good reliability (AUC 0.69-0.98), with performance notably stronger at higher severity thresholds. Lower extremity and trunk movements showed moderate reliability (AUC 0.75-0.84). Detection of eye movements was more challenging (AUC 0.51-0.63), likely reflecting the subtlety of these movements and their potential confounding with normal blinking patterns.

### Correlation With CTI Components

**Figure 4:**
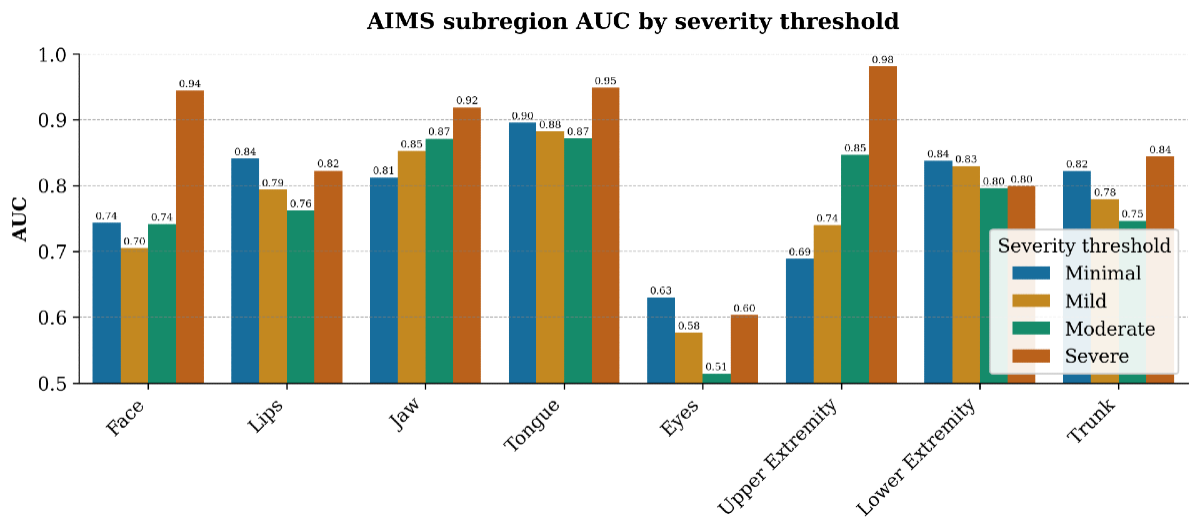
Area under the curve (AUC) across movement categories and severities. AIMS: Abnormal Involuntary Movement Scale.

As a secondary exploratory analysis, we examined how well TDtect regional subscores aligned with CTI frequency ratings to assess anatomical specificity of model predictions. Analysis of correlations with CTI frequency ratings revealed moderate-to-strong associations across movement categories (Figure 4).

Particularly noteworthy was the anatomical specificity observed in these correlations. The model’s region-specific subscores showed the strongest relationships with clinically rated movements in the corresponding body areas (Table 3). For example, the jaw subscore correlated most strongly with CTI jaw movement ratings (*r*=0.732), and the tongue subscore correlated most strongly with CTI tongue movement items (*r*=0.55). This anatomical consistency provides compelling evidence that the model is detecting clinically meaningful movement patterns rather than nonspecific indicators.

**Table 3:** Correlations between Clinician’s Tardive Inventory frequency ratings and predicted Abnormal Involuntary Movement Scale subscores.

Frequency metric^a^	Frequency value (95% CI)	Predicted subregion score^b^
Eye	0.499 (0.300-0.679)	Face
Tongue	0.552 (0.419-0.719)	Tongue
Jaw	0.732 (0.626-0.828)	Jaw
Complex	0.572 (0.393-0.730)	Upper extremity
Limb	0.261 (0.243-0.549)	Trunk
Vocal	0.464 (0.248-0.718)	Face

^a^Frequency metrics were derived from the Clinician’s Tardive Inventory examination.

^b^Subregion predicted by the model with the highest correlation.

## Discussion

### Principal Findings

The results of this study demonstrate that an artificial intelligence (AI)–powered video analysis model can provide reliable TD assessments, even in the absence of a standard video capture protocol. This cross-dataset evaluation suggests that automated screening may complement clinician assessments, supporting further investigation into its role in clinical workflows.

The AUC of 0.854, using the Schooler-Kane criteria, highlights the difficulty of distinguishing TD from non-TD in a population in which about 80% of participants meet diagnostic criteria—a class imbalance that narrows the range of possible AUC values compared with a balanced population. When evaluated at a threshold that provides a more balanced class split (AIMS ≥12; 36% positive cases), the model achieves an AUC of 0.900, closely aligning with the 0.89 AUC reported in earlier validation studies conducted under structured AIMS protocols. The remaining performance difference at the Schooler-Kane threshold is expected, considering that the model was applied without any fine-tuning to an unstructured video protocol on which it was not trained; temporal averaging of frame-level predictions was the only adjustment. Fine-tuning on a larger unstructured dataset could potentially reduce this gap further.

The moderate performance in functional impairment categories (*r*=0.27-0.63) is notable, given that the model was trained exclusively on motor movement data without functional impact labels, suggesting that the algorithm captures movement patterns that genuinely predict real-world consequences. Future iterations may improve these predictions by incorporating additional training datasets and refined subregion analysis.

The observed pattern of anatomical specificity in our correlations has important implications. The higher reliability in orofacial regions (particularly tongue and jaw movements) aligns with the typical presentation of TD, in which these movements are often the earliest and most persistent manifestations. The comparatively lower correlations with limb movements (*r*=0.261) suggest that additional training may be needed to recognize the more variable presentation of TD in the extremities. Importantly, the strong agreement between the model and clinician ratings for jaw movements (*r*=0.732) is particularly promising, as these movements are often considered diagnostic hallmarks of TD and disabling for patients. The model’s ability to detect these movements with high accuracy, even in nonstandardized video recordings, suggests that it could serve as an effective screening tool in real-world clinical settings (eg, accessing a video collected during a routine telemedicine appointment) where structured assessments may not always be practical.

### Remote Monitoring and Specialist Integration

These results support the exploration of TDtect’s integration into remote monitoring workflows. Implementing such an envisioned remote patient monitoring service could represent a paradigm shift in TD management. The AI-based detection model could be integrated into both traditional and telepsychiatry practice, allowing psychiatrists to monitor patients remotely. When TD is detected, the patient’s case could be escalated to a movement disorder specialist for confirmation; treatment recommendations; and, if needed, ongoing management. A structured referral system, as has proven successful in other disorders that require high degrees of expertise, might ensure that patients receive timely and expert intervention. Future work should evaluate whether AI-flagged cases can be effectively escalated to movement disorder specialists and whether such workflows improve early detection rates in real-world settings.

TDtect is designed as a screening tool; its intended clinical role is to identify patients who may have TD and should be referred for formal evaluation, not to replace clinician-administered diagnostic instruments such as the AIMS or CTI. Performance metrics in this paper should be interpreted in this context: sensitivity at the detection threshold is the primary concern, proportional bias at higher severity levels is a secondary consideration relevant to future severity quantification work, and all claims about clinical deployment refer to screening workflows in which positive flags are followed by clinician confirmation.

### Limitations

Although TDtect demonstrated high accuracy in detecting TD, several limitations must be acknowledged, suggesting areas for further investigation and clinical development. First, the current model does not yet provide automated assessment of frequency and severity across all movement zones defined by the CTI; future iterations should be trained to generate severity and frequency ratings across the full spectrum of clinically relevant abnormal movements. The robustness of diagnostic predictions remains constrained by the size and diversity of the training dataset, highlighting the need to incorporate broader and more representative real-world clinical data. Additionally, rigorous prospective trials remain necessary to clinically validate the TDtect algorithm, particularly through comparative studies evaluating its severity assessments against consensus ratings from multiple clinicians using established measures such as the AIMS and CTI. To enhance the model’s differential diagnostic capacity, future training sets should integrate video data from individuals presenting with a range of other movement disorders. Personalized dynamic monitoring systems that can adapt to individual patient profiles warrant further integration, allowing for the development of actionable, patient-specific alerts and more responsive longitudinal tracking.

Small validation samples are a known limitation in biomedical reliability research. Guidelines usually recommend larger cohorts than what a single-site specialty population can realistically provide for stable agreement estimates [19,20]. Our evaluation sample (N=69) was sufficient to demonstrate cross-protocol generalization, but it limits the precision of regional performance estimates, as seen in the wide bootstrap CIs for several anatomical regions, and does not permit formal demographic subgroup analysis. Data balancing and generative augmentation have shown promise in similar small, imbalanced clinical datasets [21], though augmenting the evaluation set alone would not fix the fundamental constraint here. Collecting larger unstructured video datasets prospectively remains the most direct way to refine the temporal aggregation process and achieve more accurate generalizability estimates.

Interpretation of TDtect’s performance is also limited by inherent differences in protocol across the CTI dataset, which may pose challenges for direct comparison with standardized rating instruments. Furthermore, the absence of model fine-tuning with this dataset suggests that further targeted training may yield higher accuracy. Ultimately, by addressing these limitations—including refining sensitivity thresholds, expanding the training corpus, and advancing model personalization—the algorithm may evolve into a more comprehensive AI-driven diagnostic and monitoring platform, bridging the gap between automated detection and specialist intervention [12,13].

The dataset’s high TD prevalence (approximately 80% positive under the Schooler-Kane criteria) reflects the specialty movement disorder population from which it was drawn, but limits generalizability to screening contexts where prevalence is lower. AUC values should be interpreted in light of this class distribution; positive and negative predictive values would differ substantially in a general psychiatric population. The AIMS ≥12 threshold result (AUC 0.900; 36% positive for likelihood of having TD vs 64% negative for likelihood not having TD; the CTI dataset is 80% positive for TD) provides a complementary estimate under more balanced conditions.

This analysis does not include a formal evaluation of algorithmic bias across demographic subgroups. Although the training dataset included participants across racial and ethnic groups (Sterns et al [13]), the CTI sample (N=69) does not provide sufficient statistical power for subgroup performance analysis. Future studies should incorporate larger, demographically stratified samples to evaluate potential differential performance. Additionally, although the CTI dataset’s variable recording conditions represent a step toward real-world robustness, systematic evaluation across telehealth platforms, mobile devices, and diverse environmental conditions is needed before deployment at scale.

### Future Research

Future research should emphasize a longitudinal approach in which individuals are monitored monthly or quarterly alongside medication tracking to fully evaluate the potential of smartphone-based monitoring of TD. Another avenue for future research is to explore the integration of this AI-based diagnostic tool into routine telemedicine sessions. This could enhance ongoing care for individuals receiving antipsychotic medications by providing continuous, remote monitoring for early signs of TD, allowing for timely intervention.

Future research should also focus on integrating medication adherence monitoring with TD diagnostics. Understanding patients’ adherence patterns will provide critical insights into how treatment strategies can be adjusted to prevent the progression of TD and reduce the risk of irreversible symptoms.

### Conclusions

The TDtect model demonstrates promising cross-dataset generalization in detecting TD movements when evaluated on the independent CTI dataset, with particularly robust performance in identifying anatomically specific movement patterns. Notably, the model maintained high accuracy even without standardized video capture protocols. These findings support the feasibility of automated, AI-driven movement disorder assessment in real-world clinical settings. If confirmed in prospective studies, this technology could contribute to earlier detection of TD and support more timely clinical decision-making.

## Appendix

Multimedia Appendix 1 Calibration curve for Abnormal Involuntary Movement Scale total score ≥12.

## Abbreviations

AI: artificial intelligence
AIMS: Abnormal Involuntary Movement Scale
AUC: area under the curve
CTI: Clinician’s Tardive Inventory
TD: tardive dyskinesia

## Data Availability

The datasets generated or analyzed during this study are not publicly available due fact that the video data underlying this study cannot be shared publicly due to participant privacy and institutional review board constraints, but an anonymized dataset of model predictions and clinician ratings are available from the corresponding author on request.
