# Supplementary figures and images for: Cross-Dataset Evaluation of an Automated Video-Based Model for Detecting Tardive Dyskinesia Using the Clinician’s Tardive Inventory: Validation Study
Source: JMIR Ment Health. 2026 May 14;13:e92197. doi: 10.2196/92197 (PMC13219985; doi:10.2196/92197)

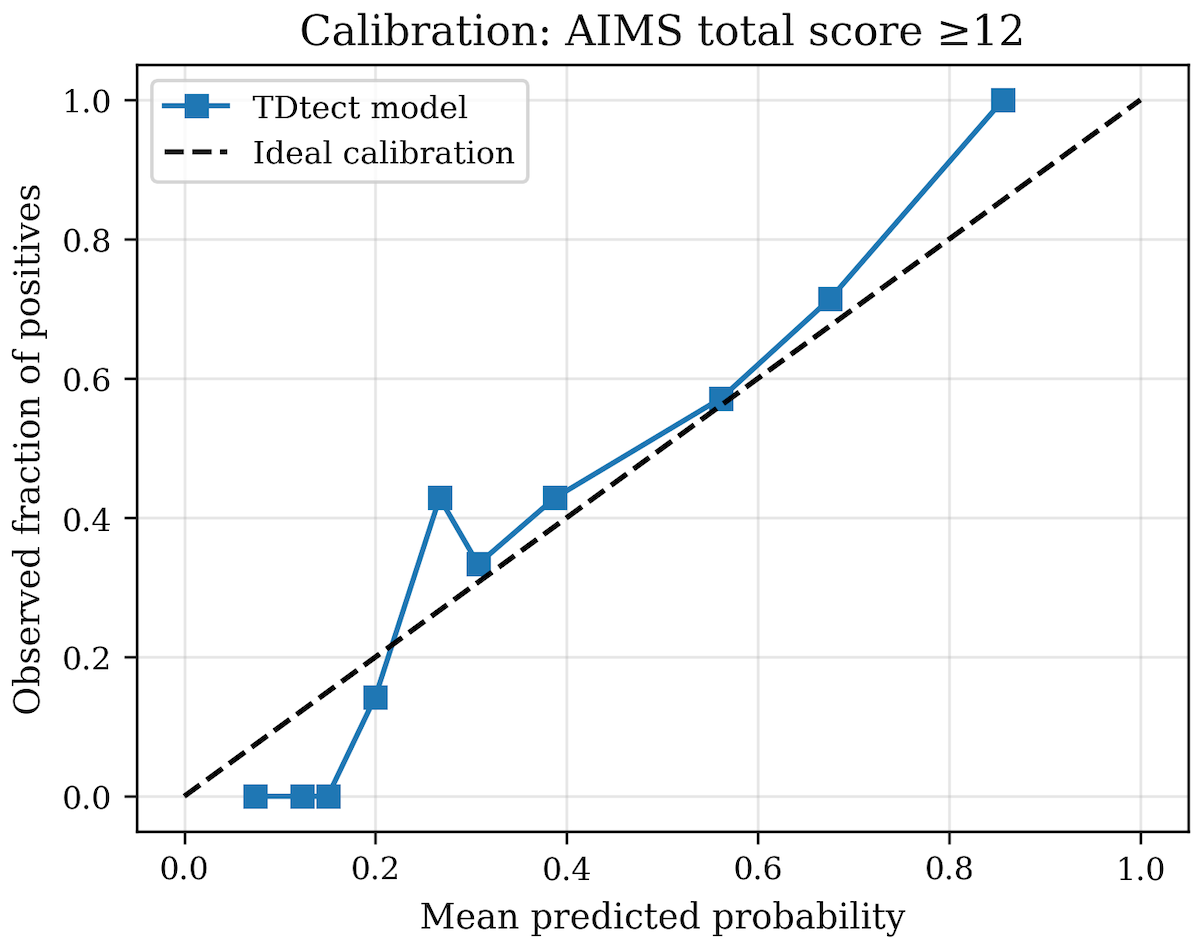

Supplement: Multimedia Appendix 1 [file mental_v13i1e92197_app1.png]
